# Angiotensin Receptor Neprilysin Inhibitor Attenuates Myocardial Remodeling and Improves Infarct Perfusion in Experimental Heart Failure

**DOI:** 10.1038/s41598-019-42113-0

**Published:** 2019-04-08

**Authors:** Daniel Pfau, Stephanie L. Thorn, Jiasheng Zhang, Nicole Mikush, Jennifer M. Renaud, Ran Klein, Robert A. deKemp, Xiaohong Wu, Xiaoyue Hu, Albert J. Sinusas, Lawrence H. Young, Daniela Tirziu

**Affiliations:** 1 Yale Cardiovascular Research Center, Section of Cardiovascular Medicine, Department of Internal Medicine, Yale School of Medicine, New Haven, CT USA; 2 Yale Translational Research Imaging Center, Section of Cardiovascular Medicine, Department of Internal Medicine, Yale School of Medicine, New Haven, CT USA; 3 University of Ottawa Heart Institute, Ottawa, Ontario Canada; 4 Department of Radiology & Biomedical Imaging, Yale School of Medicine, New Haven, CT USA; 5 Yale Cardiovascular Research Group, Section of Cardiovascular Medicine, Department of Internal Medicine, Yale School of Medicine, New Haven, CT USA; 6 Division of Nuclear Medicine, Department of Medicine, University of Ottawa, Ottawa, Ontario Canada

## Abstract

Angiotensin receptor blocker-neprilysin inhibitor (ARNi) therapy improves the prognosis of heart failure patients. However, the mechanisms remain unclear. This study investigated the biological effects of ARNi with neprilysin inhibitor sacubitril and angiotensin receptor blocker valsartan on myocardial remodeling and cardiac perfusion in experimental heart failure (HF) after myocardial infarction (MI). Male Lewis rats (10-weeks old) with confirmed HF were randomized one-week post-MI to treatment with vehicle (water), sacubitril/valsartan or valsartan, as comparator group, for either 1 or 5 weeks. Sacubitril/valsartan for 1-week limited LV contractile dysfunction vs. vehicle and both sacubitril/valsartan and valsartan attenuated progressive LV dilation after 1 and 5 weeks treatment. After 5 weeks, both sacubitril/valsartan and valsartan reduced CTGF expression in the remote myocardium, although only sacubitril/valsartan prevented interstitial fibrosis. In the border zone, sacubitril/valsartan and valsartan reduced hypertrophic markers, but only sacubitril/valsartan reduced cardiomyocyte size and increased VEGFA expression. In the infarct, sacubitril/valsartan induced an early uptake of ^99m^Tc-NC100692 (a radiotracer of angiogenesis) and improved perfusion, as determined by ^201^Tl microSPECT/CT imaging. In conclusion, ARNi improved global LV function, limited remodeling in the remote and border zones, and increased perfusion to the infarct. Sacubitril/valsartan had more consistent effects than valsartan on LV remodeling in experimental HF.

## Introduction

The angiotensin receptor blocker-neprilysin inhibitor (ARNi) represents a new therapeutic approach in heart failure^1,2^. The PARADIGM-HF trial demonstrated significant reductions in the risk of death and heart failure hospitalization in heart failure patients with a reduced ejection fraction (HFrEF) receiving ARNi, compared to enalapril^3^. Whether ARNi treatment prevents the risk of hospitalization or death for patients with HF and preserved EF (HFpEF) remains to be determined.

ARNi, is a dual-acting crystalline complex composed of neprilysin inhibitor precursor sacubitril (SAC) and the angiotensin receptor blocker valsartan (VAL)^4,5^. After intake, SAC is cleaved into the active neprilysin inhibitor sacubitrilat^6^. Neprilysin is a neutral endopeptidase, that catalyzes the degradation of a number of vasodilator peptides, including atrial natriuretic peptide (ANP) and brain natriuretic peptide (BNP), in addition to bradykinin, substance P, and adrenomedullin and contributes to the breakdown of angiotensin II^7^. Although, the neprilysin inhibition increases the concentration of vasodilating peptides as expected, it also increases the concentration of angiotensin II and endothelin I^8^. Therefore, the neprilysin inhibition alone has little, if any, antihypertensive effect^8^. Besides promoting vasodilation, natriuretic peptides, ANP and BNP counteract cardiomyocyte hypertrophy and cardiac fibrosis and induce angiogenesis^1,9,10^. Thus, combined inhibition of neprilysin and the angiotensin II receptor has potentially synergistic actions to prevent multiple mechanisms of pathological cardiac remodeling, while increasing perfusion and angiogenesis.

In this study we investigated an experimental model of heart failure induced by myocardial infarction (MI) and applied innovative imaging technologies to assess *in vivo* pathophysiology. We tested the hypothesis that SAC/VAL in HF attenuates myocardial remodeling and improves cardiac perfusion. We utilized serial echocardiography, cellular and molecular analyses for hypertrophy, fibrosis and angiogenesis, and dual isotope microSPECT/CT imaging with NC100692, an α_v_β_3_ targeted ^99m^Tc-labeled radiotracer of angiogenesis, and ^201^Tl for the *in vivo* assessment of myocardial perfusion.

## Results

### Cardiac function and remodeling

To investigate whether SAC/VAL attenuates myocardial remodeling and improves cardiac perfusion we employed an experimental model of HF post MI induced by permanent ligation of the left anterior descending (LAD) coronary artery in rats. Permanent coronary artery ligation is an accepted model for investigating pathophysiology of chronic heart failure^11^, and we chose this model rather than ischemia-reperfusion in order to avoid the effects of drug on myocardial injury. One week following LAD ligation, rats were assessed by echocardiography for study inclusion. Rats with discernable regional wall motion abnormalities from at least the mid anterior wall to apex were randomly assigned into one of three treatments groups: Vehicle (water), VAL and SAC/VAL. All rats (n = 27–30 per treatment group) underwent serial echocardiography at both 1 week after MI and 1 week after treatment was begun. A smaller number (n = 12–13 per treatment group) were administered an additional 4 weeks of therapy and echocardiography was performed again (Supplementary Fig. S1). Treatments were administered daily in the morning (8–9 am) by oral gavage for 1 or 5 weeks. SAC/VAL (1/1 ratio) was administered at a dose of 68 mg/kg/day (68 mg: 31 mg SAC, 31 mg VAL, 6 mg sodium/water) based on prior preclinical studies defining its pharmacokinetics and pharmacodynamics^4,5^ and effects on post-MI remodeling in rats^12,13^. VAL was administered at the same dose (31 mg/kg/day) in order to provide comparable AT1 receptor inhibition to that achieved with the SAC/VAL dose.

**Figure 1:**
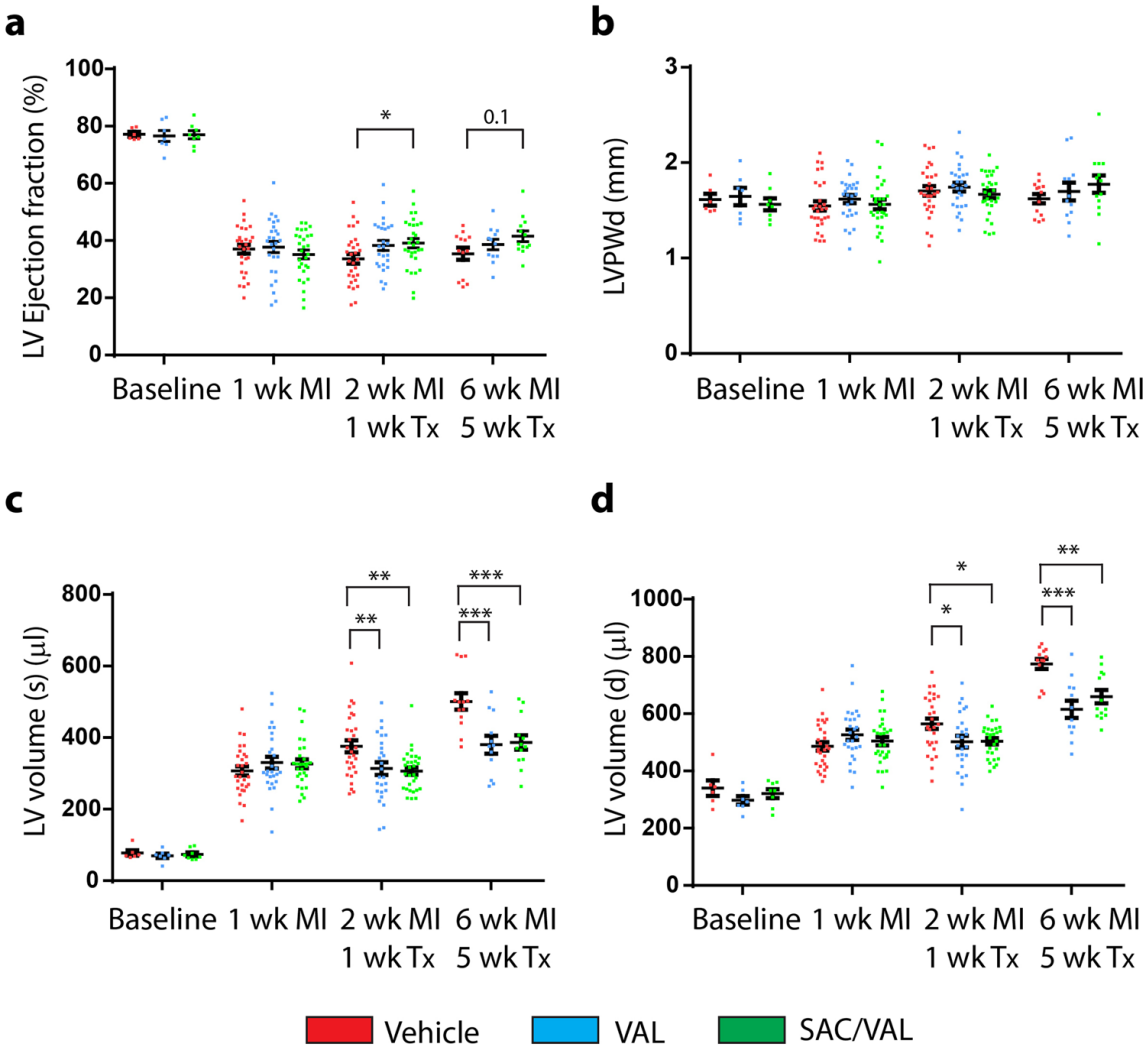
Cardiac function and remodeling. (**a**) SAC/VAL treatment increased LV Ejection fraction post MI as compared with vehicle treated rats. (**b**) No significant changes in LV posterior wall thickness in diastole (LVPWd) post MI between treatment groups. (**c**) SAC/VAL and VAL treatment reduced LV volume in systole post MI compared to vehicle treated group. (**d**) SAC/VAL and VAL treatment reduced LV volume in diastole post MI compared to vehicle treated group. Changes in LVEF, LV volume (d) and LV volume (s) were statistically significant vs. baseline in all groups. *1 and 2 wk*. *MI*/*1 wk*. *Tx*: Vehicle n = 29, VAL n = 27, SAC/VAL n = 30. *1*, *2 and 6 wk*. *MI/5 wk*. *Tx*: Vehicle n = 13, VAL n = 12, SAC/VAL n = 13. Statistical significance determined by repeated measures two-way ANOVA followed by Tukey’s post-hoc for multiple comparisons. *P < 0.05, **P < 0.01, ***P < 0.001 as indicated.

Rats randomized to treatment had a significant decrease in left ventricle ejection fraction (LVEF), from a baseline value of 75 ± 1% to 37 ± 1%, as assessed by 2D long-axis echocardiographic imaging (Fig. 1a). Following one week of treatment, the plasma ANP concentrations were numerically, but not statistically higher in SAC/VAL treated rats compared with vehicle-treated rats (1933 ± 334 pg/ml vs. 1199 ± 222 pg/ml; *P* = 0.2). In VAL treated group the ANP concentration was comparable with that in vehicle-treated rats (1346 ± 203 pg/ml, *P* = 0.9). At one week after treatment, LVEF was higher in SAC/VAL vs. vehicle treated group (39 ± 2% vs. 34 ± 2%, *P* < 0.05) and trended higher after 5 weeks (42 ± 2% vs. 35 ± 2%, *P* = 0.1) (Fig. 1a). In contrast, VAL treated rats did not demonstrate a significant increase in LVEF after either 1 week (38 ± 2%, *P* = 0.1) or 5 weeks of treatment (39 ± 2%, *P* = 0.6) (Fig. 1a). There were no statistically significant changes in the remote LV posterior wall thickness between groups, as assessed by echocardiography (Fig. 1b).

There was a marked LV dilation after MI in the vehicle treated group, with a 6-fold increase in LV systolic volume (Fig. 1c) and a 2-fold increase in LV diastolic volume (Fig. 1d) at 6 weeks post MI. VAL and SAC/VAL, both significantly blunted the progressive LV dilation after MI to a similar extent at both 1 and 5 weeks of treatment (Fig. 1c,d).

### Myocardial ^99m^Tc-NC100692 uptake and ^201^Tl perfusion

**Figure 2:**
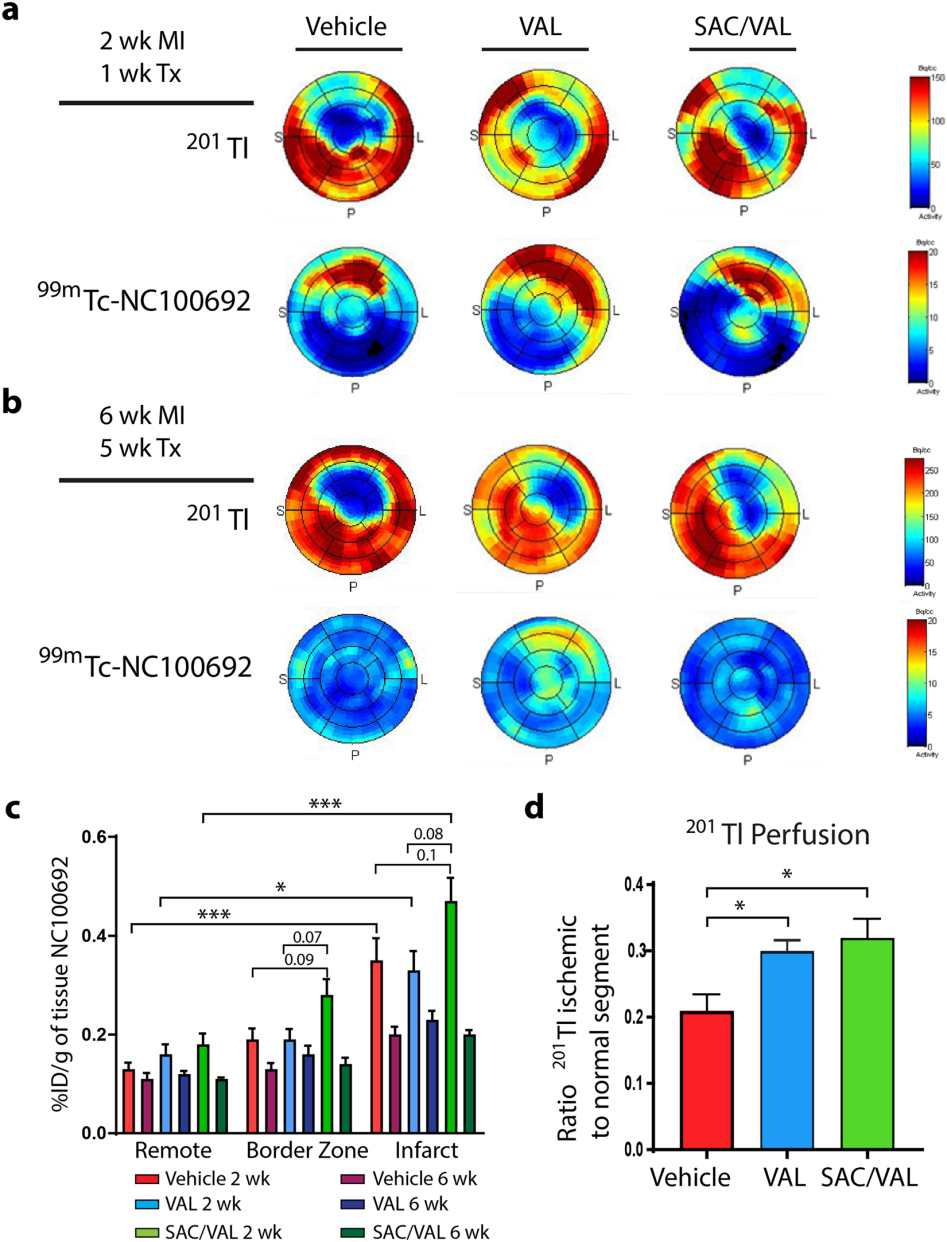
Myocardial ^99m^Tc-NC100692 uptake and ^201^Tl perfusion. (**a**,**b**) Representative *in vivo* polar-maps displayed for the left ventricle with an uptake color scale (red high, blue low) in a two dimensional image with the apex in the center and the anterior, septal, posterior, and lateral wall in a counter-clockwise formation for the perfusion tracer ^201^Tl and concurrent uptake by the angiogenesis α_v_β_3_ tracer ^99m^Tc-NC100692 at 2 wk. MI/1 wk. Tx in panel (a) and 6 wk. MI/5 wk. Tx in panel (b) for each treatment group. (**c**) Post mortem gamma well counting of ^99m^Tc-NC100692 in remote (non-infarcted myocardium), border zone, and infarct. *2 wk*. *MI/1 wk*. *Tx*: Vehicle n = 6, VAL n = 5, SAC/VAL n = 6; *6 wk*. *MI/5 wk*. *Tx*: Vehicle n = 6, VAL n = 6, SAC/VAL n = 6. (**d**) Dobutamine stress ^201^Tl perfusion. *In vivo* ^201^Tl ischemic to ^201^Tl non-ischemic under dobutamine-induced stress 6 weeks after MI. Both SAC/VAL and VAL increased dobutamine-stimulated flow to the infarct area 6 weeks after MI compared with vehicle treated rats. Vehicle n = 6, VAL n = 6, SAC/VAL n = 6. Statistical significance determined by one-way ANOVA followed by Tukey’s post-hoc for multiple comparisons. *P < 0.05, ***P < 0.001 as indicated.

To assess the effect of treatment on myocardial angiogenesis and perfusion post MI, rats were injected with ^99m^Tc-NC100692 and ^201^Tl for *in vivo* dual isotope microSPECT/CT imaging. These *in vivo* images demonstrated an increase in the uptake of angiogenesis marker ^99m^Tc-NC100692 within infarct territory (as defined by the ^201^Tl perfusion defect) in all groups at 2 weeks post MI (1 week of treatment), but not at 6 weeks post MI (5 weeks of treatment). There was no differential effect of any treatment on angiogenesis detected by *in vivo* imaging of relative ^99m^Tc-NC100692 uptake (Fig. 2a,b). We also performed *ex vivo* gamma well counting of the myocardium tissue which revealed a 2-fold increase in ^99m^Tc-NC100692 uptake in the infarct relative to the remote myocardium in all groups at 2 weeks post MI (1 week of treatment) (Fig. 2c). When compared with the vehicle and VAL groups, in the SAC/VAL group, there was a trend towards greater ^99m^Tc-NC100692 uptake by 40% (*P* = 0.1 vs. vehicle; *P* = 0.08 vs. VAL) in the infarct zone and 50% (*P* = 0.09 vs. vehicle; *P* = 0.07 vs. VAL) in the border zone. However, by 6 weeks post MI (5 weeks of treatment), there was no significant increase in ^99m^Tc-NC100692 uptake in the infarct zone compared to the remote region, and no significant differences between groups were observed (Fig. 2c).

To investigate the physiologic impact on myocardial perfusion, peak blood flow was assessed by measuring ^201^Tl uptake in rats during dobutamine stress at 6 weeks after MI (5 weeks of treatment). When compared with vehicle treated rats, both SAC/VAL and VAL groups demonstrated a significant increase in dobutamine-stimulated flow to the infarct area by 52% (*P* < 0.05) and 43% (*P* < 0.05), respectively (Fig. 2d).

### LV structural remodeling

**Figure 3:**
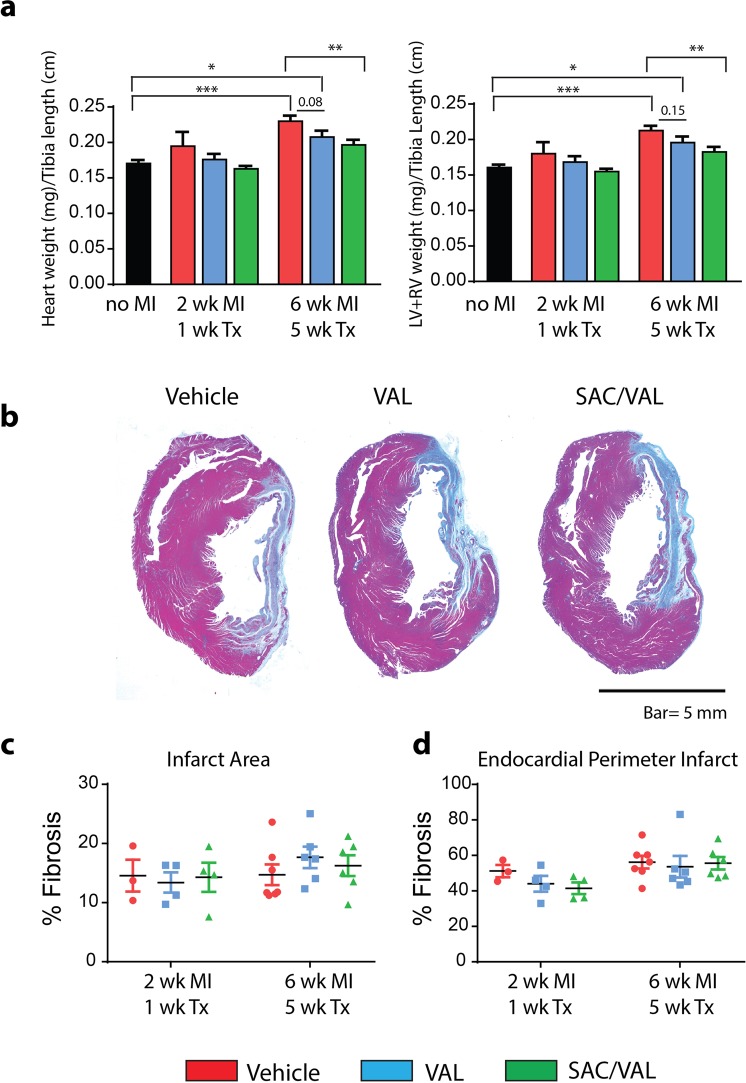
LV structural remodeling. (**a**) Total heart weights and combined left and right ventricle weights of freshly excised hearts normalized to tibia length. SAC/VAL significantly reduced the heart remodeling 6 weeks after MI compared with vehicle treated rats. (**b**) Representative Masson’s trichrome stained heart sections from vehicle, VAL and SAC/VAL treated rats at 6 wk. MI/5 wk. Tx. (**c**) Infarct size assessed on cross sections of the mid-ventricle stained with Masson’s trichrome expressed as a percent of total tissue. (**d**) Endocardial perimeter infarct expressed as a percent of total endocardial perimeter. No MI (n = 4); *2 wk*. *MI/1 wk*. *Tx*: Vehicle n = 3, VAL n = 4, SAC/VAL n = 4; *6 wk*. *MI/5 wk*. *Tx*: Vehicle n = 7; VAL n = 6, SAC/VAL n = 6–7. Statistical significance determined by one-way ANOVA followed by Tukey’s post-hoc for multiple comparisons. *P < 0.05, **P < 0.01, ***P < 0.001 as indicated.

Total heart weight and combined LV and RV weights were measured in excised hearts and normalized to tibial length (TL). There was a significant LV remodeling after MI, with a HW/TL ratio in vehicle treated rats 35% higher (*P* < 0.001) than rats not subjected to MI (no MI) (Fig. 3a). SAC/VAL treated rats demonstrated significantly lower HW/TL and LV + RV/TL values compared with vehicle treated rats (*P* < 0.01), with parallel, but non-significant trends in VAL treated rats (Fig. 3a).

Histological assessments were carried out using Masson’s trichrome staining to define the scar in cross sections from the mid LV (Fig. 3b). Mid-ventricular sections showed mild, but not statistically significant expansion of the infarct region between 2 and 6 weeks after MI, with comparable infarct sizes across all treatment groups at each time point (Fig. 3c,d).

### Interstitial fibrosis in the remote myocardium

**Figure 4:**
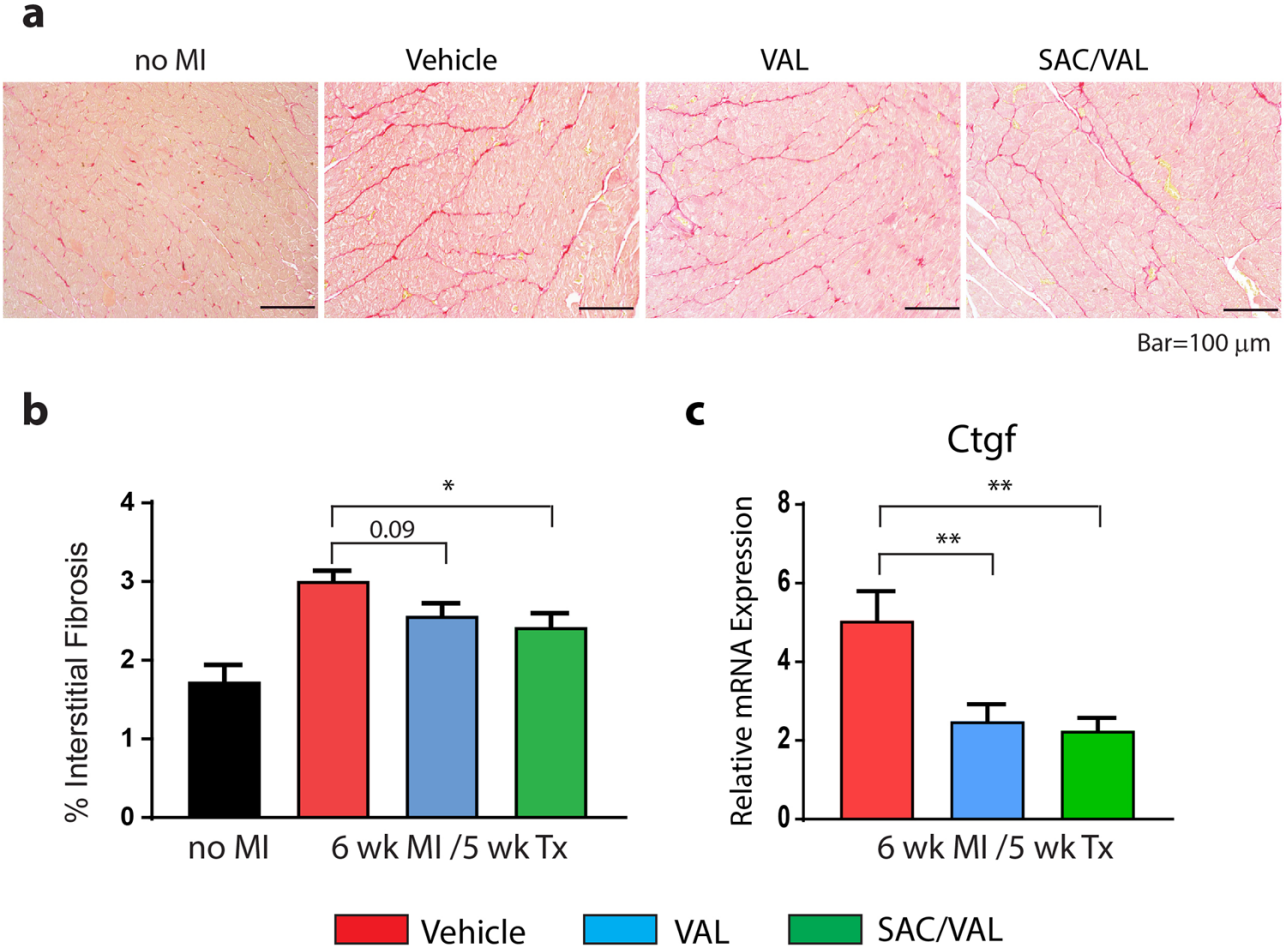
Interstitial fibrosis in the remote, non-infarcted myocardium 6 weeks after MI. (**a**) Representative images of Sirius Red staining in the remote myocardium in no MI control rats and vehicle, VAL or SAC/VAL treated rats at 6 wk. MI/5 wk. Tx. (**b**) Quantification of interstitial fibrosis. SAC/VAL significantly reduced interstitial fibrosis 6 weeks after MI, as compared with vehicle treated rats. (**c**) Both SAC/VAL and VAL significantly reduced Ctgf gene expression in remote myocardium 6 weeks after MI, as compared with vehicle treated group. No MI n = 4, Vehicle n = 7, VAL n = 6, SAC/VAL n = 7. Statistical significance determined by one-way ANOVA followed by Tukey’s post-hoc for multiple comparisons. *P < 0.05, **P < 0.01 as indicated.

Sirius Red staining of heart sections was performed to assess interstitial fibrosis in the non-infarcted, remote myocardium (Fig. 4a). There was a 74% increase in myocardial interstitial fibrosis in vehicle treated rats 6 weeks after MI, as compared with no-MI hearts (Fig. 4b). SAC/VAL treatment significantly (*P* < 0.05) prevented interstitial fibrosis in the remote myocardium when compared with vehicle treated rats. A similar effect to prevent interstitial fibrosis was observed with VAL treatment, but it was not statistically significant (*P* = 0.09) (Fig. 4b).

Connective tissue growth factor (CTGF) mediates cardiac fibrosis and extracellular matrix deposition and its expression is upregulated during cardiac remodeling^14^. To determine whether CTGF expression was affected by treatment, the Ctgf transcript levels were measured in myocardial homogenates from the remote, non-infarcted myocardium. Both VAL and SAC/VAL significantly reduced Ctgf gene expression by 50% when compared to vehicle treated rats (Fig. 4c).

### Cardiomyocyte hypertrophy and capillary/myocyte ratio in the remote myocardium

**Figure 5:**
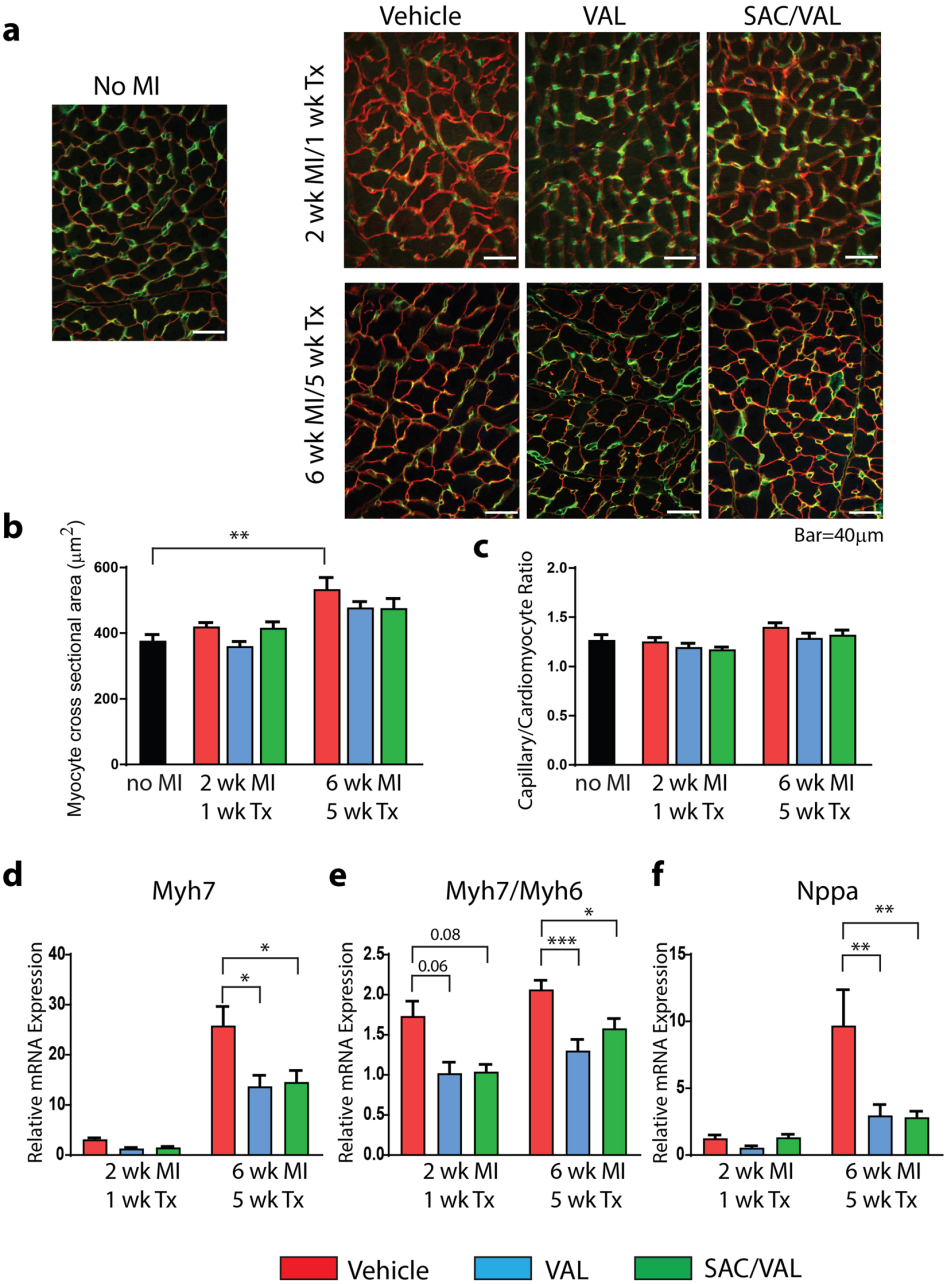
Cardiomyocyte hypertrophy and capillary/myocyte ratio in the remote, non-infarcted myocardium. (**a**) Representative images of LV sections co-stained with laminin (in red) and isolectin B4 (in green) in no MI control rats and vehicle, VAL and SAC/VAL treated rats at 2 wk. MI/1 wk. Tx and 6 wk. MI/5 wk. Tx. (**b**) Quantification of myocyte cross sectional area. VAL and SAC/VAL reduced the hypertrophic response at 6 wk. MI/5 wk. Tx. (**c**) Capillary to myocyte ratio in no MI control rats and vehicle, VAL and SAC/VAL treated rats at 2 wk. MI/1 wk. Tx and 6 wk. MI/5 wk. Tx. (**d**) SAC/VAL and VAL significantly reduced Myh7 mRNA transcript levels at 6 wk. MI/5 wk. Tx compared to vehicle treated rats. (**e**) SAC/VAL and VAL significantly reduced Myh7/Myh6 ratio at 6 wk. MI/5 wk. Tx. (**f**) SAC/VAL and VAL significantly reduced Nppa mRNA transcript levels at 6 wk. MI/5 wk. Tx. No MI n = 4; *2 wk*. *MI/1 wk*. *Tx*: Vehicle n = 3, VAL n = 4, SAC/VAL n = 4; *6 wk*. *MI/5 wk*. *Tx*: Vehicle n = 7; VAL n = 6, SAC/VAL n = 7. Statistical significance determined by one-way ANOVA followed by Tukey’s post-hoc for multiple comparisons. *P < 0.05, **P < 0.01, ***P < 0.001 as indicated.

Confocal microscopy of heart sections stained with laminin and isolectin B4 was performed to assess cardiomyocyte cross sectional area and capillary density (Fig. 5a). By 6 weeks post MI, there was a 42% increase (*P* < 0.01) in myocyte cross sectional area in the remote, non-infarcted myocardial region in vehicle treated rats compared to no-MI control rats (Fig. 5b). Treatment with VAL or SAC/VAL for 5 weeks blunted the hypertrophic response with a 27% increase of myocyte area in VAL and 26% increase in SAC/VAL when compared with no-MI controls. There were no statistically significant differences in the capillary/myocyte ratio in the remote, non-infarcted region between groups at either time point (Fig. 5c).

Further qPCR analysis was performed to assess hypertrophic markers associated with pathological remodeling in the remote myocardium. SAC/VAL or VAL treatment for 5 weeks significantly reduced the expression of β-myosin heavy chain (Myh7) by 50%, compared with the vehicle treated group (Fig. 5d). Consistent with a reduced hypertrophic response, the beta/alpha myosin heavy chain (Myh7/Myh6) ratio was significantly lower in both VAL and SAC/VAL groups after 5 weeks of treatment compared to vehicle treated rats (Fig. 5e). Similarly, SAC/VAL or VAL treatment for 5 weeks significantly reduced ANP (Nppa) transcript levels by 60% when compared to vehicle treated rats (Fig. 5f).

### Cardiomyocyte hypertrophy and angiogenesis in the border zone

**Figure 6:**
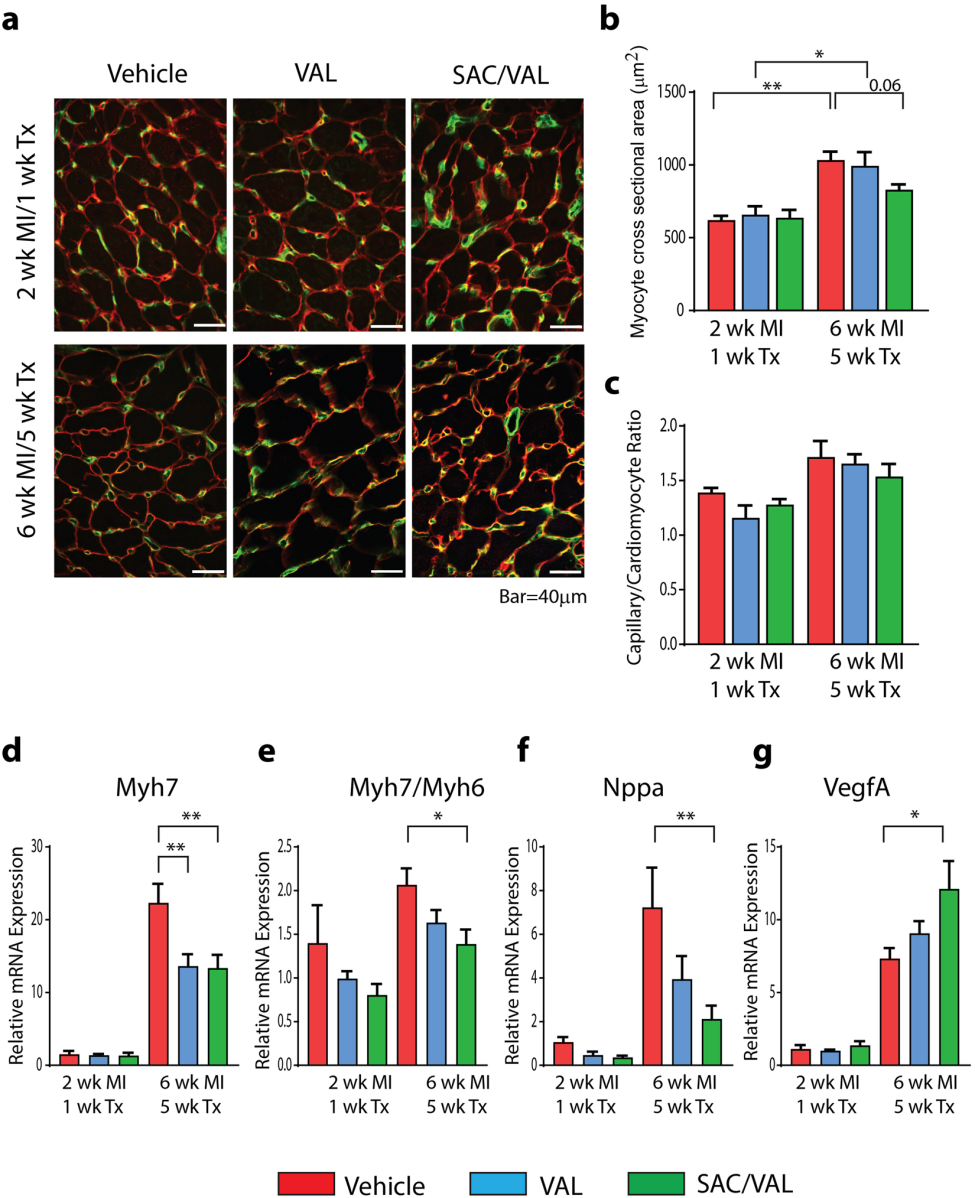
Cardiomyocyte hypertrophy and angiogenesis in the border zone. (**a**) Representative images of LV sections co-stained with laminin (in red) and isolectin B4 (in green) in vehicle, VAL or SAC/VAL treated rats at 2 wk. MI/1 wk. Tx and 6 wk. MI/5 wk. Tx. (**b**) Quantification of myocyte cross sectional area. SAC/VAL attenuated the cardiomyocyte hypertrophic response in the border zone at 6 wk. MI/5 wk. Tx, as compared with vehicle treated rats. (**c**) Capillary to myocyte ratio in vehicle, VAL or SAC/VAL treated rats at 2 wk. MI/1 wk. Tx and 6 wk. MI/5 wk. Tx. (**d**) SAC/VAL and VAL significantly reduced Myh7 mRNA transcript levels at 6 wk. MI/5 wk. Tx in the border zone. (**e**) SAC/VAL significantly reduced Myh7/Myh6 ratio at 6 wk. MI/5 wk. Tx compared to vehicle treated rats. (**f**) SAC/VAL significantly reduced Nppa mRNA transcript levels at 6 wk. MI/5 wk. Tx compared to vehicle treated rats. (**g**) Increased VegfA expression at 6 wk. MI with 5 wk. SAC/VAL treatment. *2 wk*. *MI/1 wk*. *Tx*: Vehicle n = 3, VAL n = 4, SAC/VAL n = 4; *6 wk MI/5 wk*. Tx: Vehicle n = 7; VAL n = 6, SAC/VAL n = 7. Statistical significance determined by one-way ANOVA followed by Tukey’s post-hoc for multiple comparisons. *P < 0.05, **P < 0.01 as indicated.

Substantial myocyte hypertrophy was observed in the border zone after MI (Fig. 6a). Myocyte cross sectional area increased significantly by 60% between 2 and 6 weeks after MI in both vehicle and VAL treated groups (Fig. 6b). However, in SAC/VAL the myocyte cross sectional area trended lower when compared with vehicle treated rats (*P* = 0.06) (Fig. 6b). No statistically significant differences in the capillary/myocyte ratios were observed between treatment groups at either time point (Fig. 6c). However, cardiomyocyte hypertrophy observed at 6 weeks post-MI in the border zone was accompanied by an increase in capillary density in all groups. When compared with the remote zone (Fig. 5c) the capillary/myocyte ratios in the border zone at 6 weeks post-MI tended to be higher for all treatment groups: vehicle border 1.71 ± 0.14 vs. remote 1.40 ± 0.04, *P* = 0.05; VAL border 1.65 ± 0.08 vs. remote 1.29 ± 0.05, *P* < 0.01; SAC/VAL border 1.54 ± 0.11 vs. remote 1.32 ± 0.05, *P* = 0.06. The increase in the angiogenic tracer uptake response and capillary density combined with the attenuation of cardiomyocyte growth observed with SAC/VAL treatment likely contributed to increased myocardial perfusion (shown in Fig. 2d) and improved delivery of nutrients and oxygen to infarcted myocardium.

At 5 weeks of treatment, qPCR analysis revealed significantly lower levels of Myh7 transcripts in the border zone in both VAL and SAC/VAL treated rats compared to vehicle treated rats (Fig. 6d). However, SAC/VAL, but not VAL significantly reduced the Myh7/Myh6 ratio (Fig. 6e) and Nppa gene expression (Fig. 6f). In addition, SAC/VAL treatment for 5 weeks significantly increased vascular endothelial growth factor A (VegfA) expression in the border zone, compared with vehicle treated rats (Fig. 6g).

### Vasculature in the infarcted myocardium

**Figure 7:**
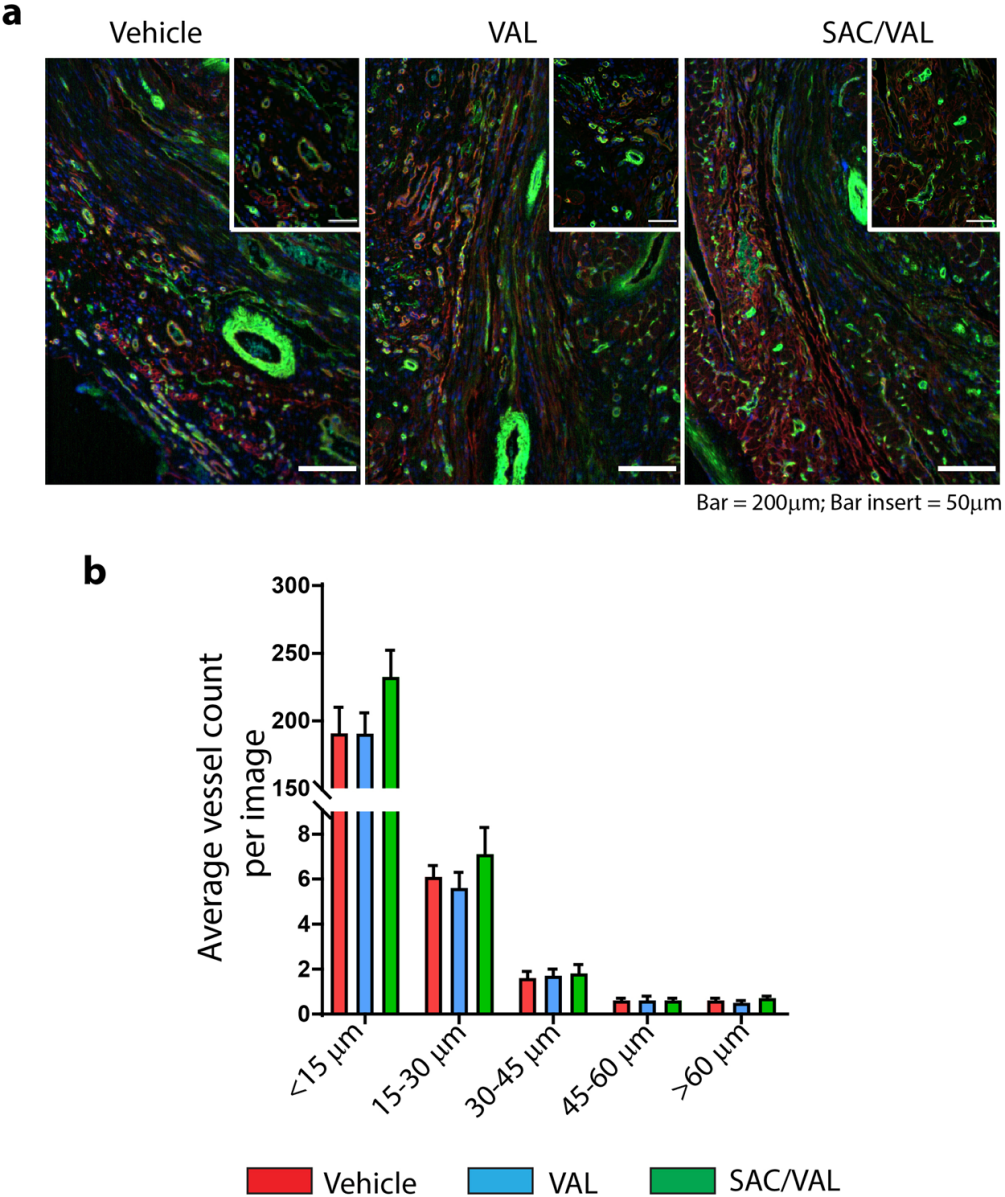
Vessel remodeling in the infarct region at 6 weeks post MI. (**a**) Representative images of infarct region co-stained with laminin (in red), isolectin B4 (in green), and DAPI (in blue) in vehicle, VAL and SAC/VAL treated rats at 6 wk. MI/5 wk. Tx. (**b**) Quantification of vessel density by vessel diameter. Values expressed as average vessel count per image. Vehicle n = 7, VAL n = 6, SAC/VAL n = 7.

Analysis of lectin staining within the infarct after 5 weeks of treatment (Fig. 7a) revealed no significant difference in the density of either the microvasculature or larger vessels between treatment groups (Fig. 7b).

## Discussion

The present study demonstrates that ARNi, SAC/VAL, limits contractile dysfunction and post-infarct pathological remodeling in a rat model of heart failure induced by permanent LAD ligation.

Our innovative imaging technologies to assess angiogenesis and blood flow revealed that SAC/VAL induced an angiogenic tracer uptake and increased perfusion in the infarct region. Furthermore, at the cellular level, SAC/VAL attenuated cardiomyocyte growth and increased capillary/cardiomyocyte ratio in the border zone and prevented myocardial fibrosis in the remote zone, while at the molecular level induced VEGFA expression in the border zone. Altogether these data demonstrate that SAC/VAL attenuates pathological remodeling in heart failure while improving myocardial perfusion and delivery of nutrients and oxygen to the infarct. In contrast, VAL had less evident effects than SAC/VAL on angiogenic tracer uptake, prevention of myocardial fibrosis, attenuation of cardiomyocyte growth and VEGFA expression. The beneficial effects of SAC/VAL were significant when compared with vehicle treated rats, and tended to be more consistent than VAL, but were not statistically different than those observed with VAL treatment in this model.

We observed beneficial effects of SAC/VAL on LV remodeling with increases in LVEF and reductions in LV volumes and mass, as compared to vehicle treated rats. Our data expand upon prior studies that also determined a significant improvement in LVEF with ARNi treatment in rats subjected to permanent LAD ligation. Of note, our model was more severe, as indicated by a much lower baseline post-MI EF (37 ± 1%) prior to treatment than in earlier studies (EF 51 ± 1% and 47 ± 2%)^12,13^. These findings are important as they indicate that SAC/VAL also has benefit in a rat model of more advanced ischemic cardiomyopathy.

The effects of SAC/VAL were not mediated by changes in infarct size, since the drug was started one week after MI induced by permanent LAD occlusion. In contrast, in a rabbit model of ischemia/reperfusion, SAC/VAL treatment begun either at the onset of reperfusion or 6 weeks later improved LVEF and reduced infarct size compared to controls, as late as 10 weeks^15,16^. In spontaneously hypertensive rats undergoing ischemia/reperfusion, SAC/VAL treatment started 4 weeks after ischemia/reperfusion prevented further decline in LVEF and reduced fibrosis in the border zone^17^. These data suggest that structural remodeling may change with SAC/VAL treatment in the context of reperfusion, even when treatment is started several weeks after initial injury. Reperfusion following occlusion may account for differences in infarct size observed across studies and may be influential in the magnitude of functional recovery observed.

Our results show that SAC/VAL prevented interstitial fibrosis in the remote, non-infarcted myocardium compared to vehicle treated rats. We also observed reductions in CTGF gene expression, a robust marker of pro-fibrotic remodeling in the heart, in both SAC/VAL and VAL treated rats, likely reflecting the inhibition of Ang II signaling^18,19^. Attenuation of interstitial fibrosis with a reduction in collagen I deposition with SAC/VAL was also reported after 4 weeks of treatment post MI in a less severe rat heart failure model^12^.

Our findings demonstrate progressive and substantial hypertrophic remodeling of surviving cardiomyocytes in the border zone after MI. SAC/VAL treatment tended to reduce border zone myocyte cross sectional area when compared with vehicle treated rats 6 weeks after MI. In contrast, VAL treatment alone appeared to have little effect. SAC/VAL treatment also led to more significant reductions in the βMHC/αMHC ratio and ANP expression in the border zone. These data demonstrate an attenuation of pathological remodeling of cardiomyocytes in the border zone post MI with SAC/VAL therapy and may have relevance to the pathogenesis of ventricular arrhythmia^20^. It is known that contractile and electrical abnormalities occur in myocytes located within the border zone^21–23^. We did not assess arrhythmia inducibility, although no rats died during the follow-up treatment period. Of interest, in the PARADIGM-HF trial, sudden cardiac death was significantly reduced by SAC/VAL compared with enalapril^24^. Further investigation is necessary to understand if ARNi results in meaningful changes in ventricular arrhythmia.

Our results indicate that SAC/VAL treatment improved late perfusion reserve within the infarct region in our rat model. We also observed increased myocardial VEGFA transcript levels in the border zone of the infarct with SAC/VAL treatment compared to vehicle treated rats. There were trends towards greater uptake of ^99m^Tc-NC100692, a radiolabeled probe that targets α_v_β_3_ integrin activation, a marker of angiogenesis, within both the border zone and infarct regions one week after SAC/VAL treatment. However, we were unable to demonstrate differences in the density of lectin-positive vasculature in the infarcts of SAC/VAL treated rats 6 weeks after MI when compared with control or VAL treated rats. Prior studies employing a rat model of permanent coronary occlusion have demonstrated that stimulation of angiogenesis early post MI results in less remodeling without an observable change in capillary density at later time points^25^. A more sustained enhancement in nitric oxide bioavailability associated with improvement in vascular function with SAC/VAL compared to VAL has been recently reported in a model of heart failure in spontaneously hypertensive rats^17^. However, in our study both SAC/VAL and VAL treated rats had comparably greater dobutamine-stimulated flow in the infarct area, as assessed by ^201^TI perfusion imaging, when compared with vehicle treated rats, suggesting improvements in vascular reserve.

Angiogenesis and preservation of blood flow are important for cardiac metabolism, which is needed to maintain contractile activity and prevent progressive LV dysfunction after MI. In the border zone, an imbalance in hypertrophic and angiogenic responses could lead to a progressive deterioration in heart function^26,27^. Whether SAC/VAL has a specific role to enhance angiogenesis remains unclear. SAC inhibits neprilysin and increases the concentrations of natriuretic peptides. Natriuretic peptides contribute to vascular regeneration in mouse models of hind limb ischemia^28,29^. In ischemic muscle and hypertrophic hearts, natriuretic peptides activate the endothelial GMP signaling to mediate the angiogenic response^9,10^. In cultured endothelial cells, ANP promotes cell proliferation and migration at low concentrations and exerts inhibitory effects at high concentrations^30^. However, it has been shown that ANP has opposing effects to negatively regulate VEGF expression by smooth muscle cells^31^ and inhibit VEGF signaling in endothelial cells^32^.

This study has some limitations. First, we did not perform hemodynamic measurements. Differential effects on preload and/or afterload might have contributed to some of the effects that we observed with these agents. Second, we have not assessed ventricular arrhythmia occurrence or inducibility, so the implications of our findings on the anti-hypertrophic effect of SAC/VAL in the border zone remain uncertain. Third, we did not define the specific mechanism through which SAC/VAL increases blood flow reserve in the infarct. Fourth, we did not use enalapril as a comparator, as was done in the PARADIGM-HF^3^ trial, so that we cannot provide insight into the differential effects of ARNi and enalapril in clinical studies. Finally, the study was not adequately powered to demonstrate statistical differences between the SAC/VAL and VAL groups.

SAC/VAL is currently investigated in several clinical trials. The PARADISE-MI evaluates SAC/VAL vs. ACE inhibitor treatment in post-AMI patients with LV systolic dysfunction in order to compare their effects on cardiovascular death and heart failure hospitalization. The PARAGON-HF trial evaluates SAC/VAL compared to VAL in patients with HFpEF in order to determine their differential effects on death and heart failure hospitalization. Preliminary results indicate that SAC/VAL reduced NT-proBNP to a greater extent than VAL alone^33^.

In conclusion, the present study provides additional insight into the effects of ARNi therapy in heart failure. In an experimental model of severe heart failure after MI, SAC/VAL had beneficial effects on LV functional and structural remodeling, limiting cardiomyocyte hypertrophy (including in the border zone) and interstitial fibrosis while increasing VEGFA expression and improving myocardial perfusion and perfusion reserve to the infarct region.

## Methods

### Surgical animal model

Myocardial infarction was induced in 8 to 10-week-old male Lewis rats by permanent ligation of the left anterior descending (LAD) coronary artery after thoracotomy under isoflurane anesthesia. The LAD was ligated 2 mm below the left atrial appendage. ECG ST segment elevation and visual observation of myocardial blanching were used to confirm coronary artery ligation. All animal experiments were performed under a protocol approved by the Institutional Animal Care and Use Committee of Yale University. All experiments were carried out in accordance with the approved guidelines.

### Study design

One week following LAD ligation, rats were assessed by echocardiography for study inclusion. Rats with discernable regional wall motion abnormalities from at least the mid anterior wall to apex were randomly assigned into one of three treatments groups: Vehicle (water, n = 29), VAL (n = 27) and SAC/VAL (n = 30). Treatments were administered daily in the morning 8–9 am, by oral gavage. SAC/VAL (1/1 ratio) was administered at a dose of 68 mg/kg/day (68 mg: 31 mg SAC, 31 mg VAL, 6 mg sodium/water). VAL was administered at the same dose (31 mg/kg/day) in order to provide comparable AT1 receptor inhibition to that achieved with the SAC/VAL dose. Within each group, rats were randomly assigned to either 1 week of treatment (Vehicle n = 16, VAL n = 15, SAC/VAL n = 17) or 5 weeks of treatment (Vehicle n = 13, VAL n = 12, SAC/VAL n = 13) and subsets of each group underwent echocardiography, hybrid SPECT/CT imaging, and histological or molecular approaches, as outlined in Supplementary Fig. S1. Color coding of the experimental groups was used to blind investigators to treatment assignment. An additional control group of 4 rats that did not undergo MI surgery (no MI) were included for molecular and histological assessments.

### Echocardiography

Serial transthoracic echocardiograms were performed prior to MI, and at 1, 2, and 6 weeks after LAD ligation. All studies were performed under light isoflurane anesthesia (1–2%) using a VisualSonics Vevo 2100 high resolution ultrasound imaging system. Two-dimensional images of the left ventricle were obtained using a 21 MHz transducer in both short axis and long axis views for determination of LV ejection fraction, LV end-diastolic and end-systolic volumes, and LV posterior wall thickness.

### Quantitative assessment of myocardial angiogenesis and perfusion with dual-isotope hybrid MicroSPECT/CT imaging

A ^99m^Tc-labeled cyclic arginine-glycine-aspartate (RGD) peptide (^99m^Tc-NC100692, Maraciclatide, GE Healthcare) that targets the activated conformation of α_v_β_3_ integrin was used to evaluate myocardial angiogenesis at both 2 and 6 weeks after MI in combination with ^201^Tl perfusion imaging. Myocardial uptake of ^99m^Tc- NC100692 early post MI has been shown to target α_v_β_3_ integrin activation on endothelial cells in sites of ischemia-induced angiogenesis post-MI^25,34^. Hybrid dual isotope microSPECT/CT imaging of ^99m^Tc- NC100692 and ^201^Tl was performed at both 2 weeks post-MI (1 week treatment) and 6 weeks post-MI (5 weeks treatment) using a high-resolution (<0.75 mm) and high-sensitivity (>700 cps/MBq) microSPECT/CT system (uSPECT-4, MILabs, Utrecht, Netherlands). Rats were injected with ^99m^Tc-NC100692 (4–6 mCi iv, via tail vein) 60–90 min prior to ^201^Tl (0.4–1.5 mCi iv) administration at rest after 1 week of treatment. Rats were also evaluated after 5 weeks of treatment using the same dual isotope imaging protocol, however, ^201^Tl was administered during graded dobutamine-induced stress. Dobutamine was infused via a secondary lateral tail vein catheter at increasing doses (5, 10 and 20 μg/kg/min) at 3 min intervals. ^201^Tl was injected at minute three of the 20 μg/kg/min dobutamine infusion, which was continued for an additional 3 min to maximize ^201^Tl uptake. All rats underwent dual isotope imaging for 30 min, and acquired images were reconstructed using MLEM iterative reconstruction with 16 iterations, 9 subsets and a voxel size of 0.4 mm. The reconstructed myocardial uptake images were assessed with FlowQuant^®^ software using an American Heart Association 20 segment model. Uptake greater than 75% of the maximum was used to delineate segments with normal perfusion, while segments with relative perfusion less than 40% of the max were classified as ischemic. The *in vivo* myocardial distributions of ^99m^Tc-NC100692 counts and ^201^Tl were evaluated qualitatively from images and bullseye maps in addition to quantitative assessment of relative myocardial perfusion reported as the ratio of ischemic to normal uptake. Following *in vivo* imaging, animals were sacrificed, hearts excised, and the LV myocardium processed *for ex* vivo gamma well counting. The LV was sectioned into infarct, border and remote zones. Tissue ^201^Tl and ^99m^Tc-NC100692 were measured with a gamma-well counter (Cobra, Packard) using appropriate energy windows (^201^Tl: 78 KeV ±5%; ^99m^Tc: 140 KeV ±5%). Raw counts were corrected for spillover, background, decay and tissue weight. Corrected counts for ^99m^Tc-NC100692 were converted to % injected dose per gram of tissue (%ID/g).

### Histological assessments

Isolated heart tissue samples were fixed in 4% paraformaldehyde and embedded in paraffin. Infarct size, interstitial fibrosis, capillary density, and cardiomyocyte cross-sectional area were assessed at both 2 weeks post-MI/1-week treatment and 6 weeks post-MI/5 weeks treatment on 5-μm sections.

Infarct size was determined on Masson’s trichrome stained heart sections imaged with a Leica M165C and analyzed using ImageJ software. Specifically, blue stained pixels (identifying scar tissue) were quantified and calculated as a percentage of all pixels (scar and myocardium); the infarct size was expressed as percentage of total heart cross sectional area, as previously described^35^. Endocardial perimeter of the infarct was also quantified using ImageJ by manually tracing the endocardium that was blue (scar), and calculated as a percentage of the total endocardial perimeter.

Interstitial fibrosis in the remote zone was identified by Sirius Red staining, imaged with a Nikon Eclipse 80i and analyzed with Image J software. Five fields (20x magnification) per heart section were analyzed.

Capillary density and cardiomyocyte size were determined on LV sections stained with isolectin B4 (ILB4; Sigma Aldrich) and an anti-laminin antibody (Sigma-Aldrich), respectively, as previously described^36,37^. Cross-sections for counting contained circular myocytes and clear round shaped capillaries. Images (30X magnification) were captured using a spinning disk Nikon Ti-E confocal microscope and analyzed with Image J software. In total, 7 fields in the LV remote zone and 3 fields in the border zone were analyzed per heart. To analyze blood vessels within the infarct, images (6x magnification) were taken along the length of the scar of each heart section. On average, 8 images within the infarct zone were analyzed per rat.

### Gene expressions of hypertrophic, fibrotic and angiogenic markers

The mRNA expression of α-myosin heavy chain (α-MHC/Myh6), β-myosin heavy chain (β-MHC/Myh7), ANP/Nppa, connective tissue growth factor (CTGF/Ctgf) and angiogenic factor (VEGFA/VegfA) were determined by quantitative PCR. Total RNA was isolated from LV samples using the RNeasy Plus Mini Kit (Qiagen) and reverse transcribed (500 ng RNA) using the iScript cDNA synthesis kit (Bio-Rad Laboratories). qPCR was carried out using iQ SYBR Green supermix kit (Bio-Rad Laboratories) in a CFX96 real-time PCR detection system (Bio-Rad Laboratories). Validated primer sets for rat Myh6, Myh7, Nppa, Ctgf, and VegfA (Qiagen) were used. The expression levels were normalized to Rpl32.

### Plasma ANP assessment

Blood samples were collected 3–5 hours after the last dose administration. ANP concentration in plasma was determined using a fluorescent immunoassay kit (Phoenix Pharmaceutical, INC).

### Statistical analysis

Data are presented as mean ± SEM. Differences between groups were assessed using one-way ANOVA, two-way ANOVA or repeated measures two-way ANOVA followed by Tukey’s or Sidak’s post-hoc test for multiple comparisons, as appropriate. Comparisons between two independent groups were performed using a two-sample t test. All *p* values were calculated using two-tailed statistical tests. Differences were considered significant when *p* < 0.05. Data were analyzed using GraphPad Prism Version 6.03 or SPSS version 24.

## Supplementary information

Supplementary Figure 1

## Data Availability

No large datasets were generated for this article. The data generated and analyzed in this study are available from the corresponding author on reasonable request.
