# Supplementary material for: Angiotensin Receptor Neprilysin Inhibitor Attenuates Myocardial Remodeling and Improves Infarct Perfusion in Experimental Heart Failure
Source: Sci Rep. 2019 Apr 8;9:5791. doi: 10.1038/s41598-019-42113-0 (PMC6453892; doi:10.1038/s41598-019-42113-0)
Supplement: Supplementary file 1 — Supplementary Figure 1 [file 41598_2019_42113_MOESM1_ESM.pdf]

SUPPLEMENTARY DATA

Angiotensin Receptor Neprilysin Inhibitor Attenuates Myocardial Remodeling and Improves Infarct Perfusion in Experimental Heart Failure

Daniel Pfau, Stephanie L. Thorn, Jiasheng Zhang, Nicole Mikush, Jennifer M. Renaud, Ran Klein, Robert A. deKemp, Xiaohong Wu, Xiaoyue Hu, Albert J. Sinusas, Lawrence H. Young, Daniela Tirziu

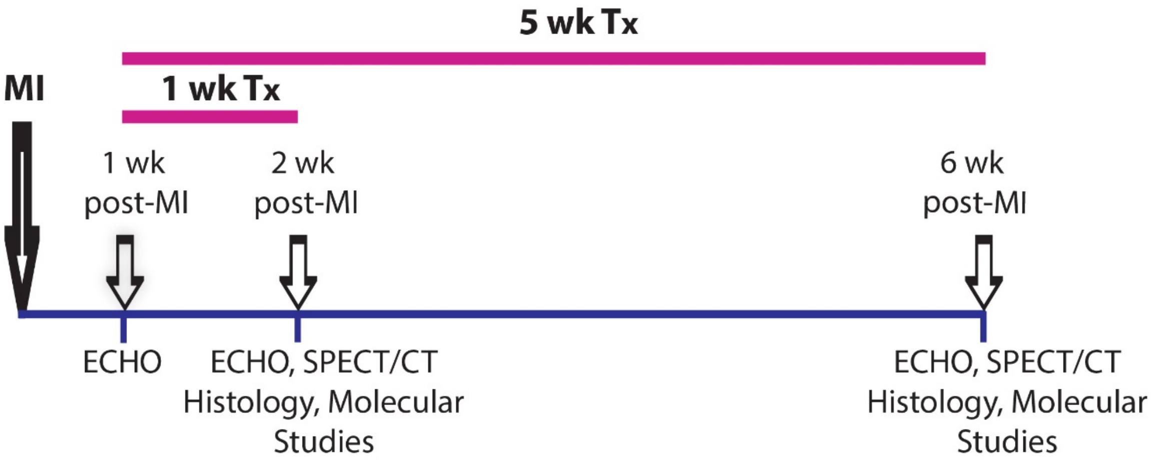

Supplementary Figure S1. Schematic representation of experimental approach. One-week post-LAD ligation, rats with confirmed MI were treated with SAC, SAC/VAL or vehicle (water). After 1 week or 5 weeks of treatment, subsets of rats were assessed by serial two-dimensional echocardiography, hybrid SPECT/CT imaging with ^{201}Tl (for myocardial perfusion) and ^{99m}Tc-NC100692 (for myocardial angiogenesis), and histological and molecular analyses.
